# Multiple forms of atypical rearrangements generating supernumerary derivative chromosome 15

**DOI:** 10.1186/1471-2156-9-2

**Published:** 2008-01-04

**Authors:** Nicholas J Wang, Alexander S Parokonny, Karen N Thatcher, Jennette Driscoll, Barbara M Malone, Naghmeh Dorrani, Marian Sigman, Janine M LaSalle, N Carolyn Schanen

**Affiliations:** 1 Department of Human Genetics, UCLA Geffen School of Medicine, Los Angeles, California, 90095, USA; 2 Nemours Biomedical Research, Alfred I. duPont Hospital for Children, Wilmington, Delaware, 19803, USA; 3 Dept. of Medical Microbiology and Immunology, University of California, Davis, California, 95616, USA; 4 Neuropsychiatric Institute, UCLA Geffen School of Medicine, University of California, Los Angeles, California, 90095, USA; 5 Department of Biological Sciences, University of Delaware, Newark, DE, 19716, USA; 6 Department of Pediatrics, Thomas Jefferson University, Philadelphia, Pennsylvania, 19107, USA

## Abstract

**Background:**

Maternally-derived duplications that include the imprinted region on the proximal long arm of chromosome 15 underlie a complex neurobehavioral disorder characterized by cognitive impairment, seizures and a substantial risk for autism spectrum disorders[1]. The duplications most often take the form of a supernumerary pseudodicentric derivative chromosome 15 [der(15)] that has been called inverted duplication 15 or isodicentric 15 [idic(15)], although interstitial rearrangements also occur. Similar to the deletions found in most cases of Angelman and Prader Willi syndrome, the duplications appear to be mediated by unequal homologous recombination involving low copy repeats (LCR) that are found clustered in the region. Five recurrent breakpoints have been described in most cases of segmental aneuploidy of chromosome 15q11-q13 and previous studies have shown that most idic(15) chromosomes arise through BP3:BP3 or BP4:BP5 recombination events.

**Results:**

Here we describe four duplication chromosomes that show evidence of atypical recombination events that involve regions outside the common breakpoints. Additionally, in one patient with a mosaic complex der(15), we examined homologous pairing of chromosome 15q11-q13 alleles by FISH in a region of frontal cortex, which identified mosaicism in this tissue and also demonstrated pairing of the signals from the der(15) and the normal homologues.

**Conclusion:**

Involvement of atypical BP in the generation of idic(15) chromosomes can lead to considerable structural heterogeneity.

## Background

**Figure 1:**
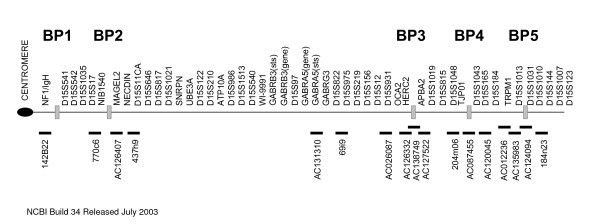
Schematic of the duplication region showing the relative positions of genes and FISH probes used (not to scale).

Chromosome 15q11-q13 is a region highly susceptible to genomic rearrangements including interstitial deletions, duplications, triplications, as well generation of supernumerary pseudodicentric chromosomes also termed isodicentric(15) or idic(15). Because the region is subject to genomic imprinting, deletions of the region lead to Prader-Willi syndrome (PWS) or Angelman syndrome (AS) depending upon the parent of origin of the deletion. The deletions generally occur using three commonly recognized breakpoints within the region (BP1-BP3)[2,3] while duplications and triplications have been described that use two additional breakpoints (BP4-5) located telomeric to BP3 (Figure 1). Similar to the deletions, parent of origin effects are evident in the phenotypes arising from duplication events[4]. The critical ~6 Mb region responsible for PWS/AS and the duplication chromosome 15 syndromes lies between BP2 and BP3[5]. Homologous pairing of this region has been observed in cycling lymphocytes [6] and cortical neurons [7].

The segmental nature of the rearrangements arises due to a series of large transcribed repeats derived from the *HERC2 *locus as well as low copy repeats of chromosome 15 (LCR15s), which provide the basis for the stereotypic nature of most rearrangements involving this region [8-10]. It is believed that the LCR cause misalignment during meiosis I, which leads to unequal but homologous recombination events involving both sister chromatid or interchromosomal exchanges[11]. In addition to the repeat-mediated illegitimate recombination events, a region with a high rate of recombination has been identified within the PWS/AS critical region. This region lies near the D15S122 and the *GABRB3 *loci, which lie ~1 Mb apart, yet show genetic distances of ~4 cM in females and ~1 cM in males, suggesting a recombination hotspot in females [12].

We recently developed an array comparative genomic hybridization (array-CGH) tool to examine duplications of chromosome 15q [13]. Combining this array with standard molecular and cytogenetic strategies, we identified four patients with atypical forms of idic15 chromosomes that lead to varying degrees of segmental aneuploidy for the proximal long arm and that indicate that additional crossovers may occur within the idic(15) chromosome.

## Results

Supernumerary der(15) chromosomes were identified in metaphase spreads for each of the probands as well as the mother in family 99.10. Samples from patients 99.30 and 03.46 carried single idic(15) chromosomes that included two centromeres identified by the centromeric probe, pcm15. The sample from patient 00.16 carries two derivative chromosomes in each cell, each of which showed also carried two signals for the centromeric probe. Neither 99.30 nor 00.16 showed evidence for mosaicism while 03.46 was mosaic with approximately 70% of PHA stimulated peripheral white blood cells carrying the idic15, and 30% showing a normal karyotype. Maternal karyotypes for each of these cases were normal. In family 99.10, the proband carried a nonmosaic idic(15) and had low level mosaicism for a large ring chromosome 15 [r(15)]. Her mother was also mosaic for the ring chromosome. Clinical characteristics of these subjects were consistent with the dup(15) phenotype and are noted in Table 1.

**Table 1:** Clinical Characteristics of subjects with atypical duplications

Subject ID	Chronological Age at examination	Autism	Mean Mental Age	Seizures	Facial Dysmorphisms
00.16	74 m	Yes	9.5 mos	Infantile spasms	Epicanthus, low nasal bridge, unfolded ears
99.30	123 m	Yes	10.75 mos	Absence seizures	Microcephaly, epicanthus
03.46	102 m	Yes	13.75 mos	Infantile spasms, Lennox Gastaut	Epicanthus, unilateral cryptorchidism
99.10	64 m	Yes	24 mos	none	Epicanthus, low nasal bridge, unfolded ears
99.12	N/A	No	N/A	none	none

## Discussion

### Patient 00.16

**Figure 2:**
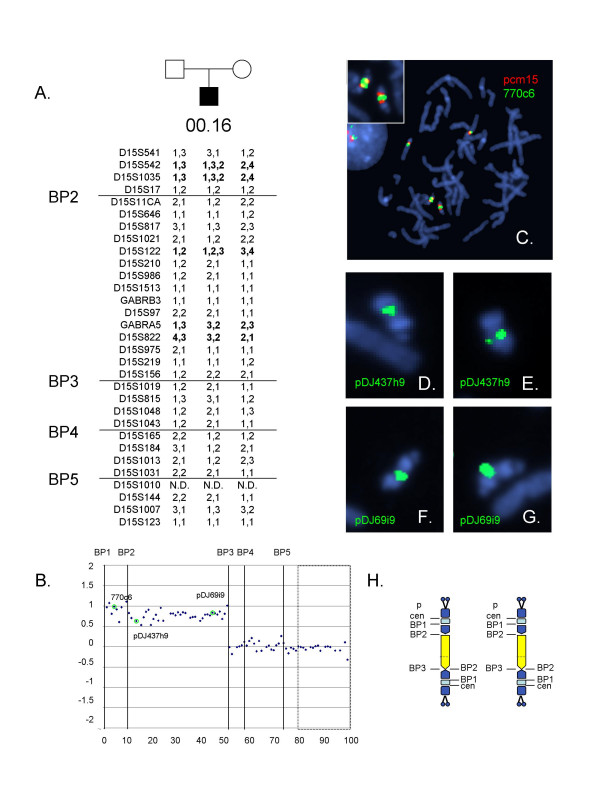
Molecular and cytogenetic data for patient 00.16. A. Genotyping analyses using STS markers spanning the duplication region reveal evidence for an interchromosomal exchange leading to the formation of the der(15). The shift from biallelic to monoallelic maternal contribution could arise from a second exchange within the duplication or from a crossover on the normal maternal homologue. B. Graph of the Log_2_T/R ratios for probes across chromosome 15q11-q14. The positions of the BP are shown as vertical lines. The dotted box indicates control probes distal to BP5 and from other autosomes. The positions of the FISH probes are indicated. The dosage data are consistent with tetrasomy of the region between BP2-BP3. C, Metaphase chromosomes hybridized with centromere probe, pcm15, and 770c6 show signals on the normal homologues as well as two centromeric signals on both der(15)s (inset). D and E. Images of idic(15) chromosomes from a single metaphase spread hybridized with pDJ437h9. F and G. Images of idic(15) chromosomes from a single metaphase spread hybridized with pDJ69i9. H. Schematic of the duplication chromosomes for subject 00.16. The der(15) der(15)s are satellited on both p arms, the heteromorphic region encompassing the NF1 pseudogene region is shown in light blue. The region between BP1-BP2 is shown in blue and present on both ends of the chromosome with a single copy of the region between BP2-BP3 shown in yellow. The approximate position of a potential crossover within the der(15) is shown by the dashed line.

The karyotype identified in clinical studies for this patient was 48,XY+idic(15)(q12;q12)+idic(15)(q12;q12), consistent with a "typical" idic(15) morphology for the two der(15) chromosomes. Genotyping analysis showed patient 00.16 carried three alleles at D15S542, D15S1035, and D15S122, consistent with maternal origin both der(15) chromosomes (Figure 2A). However, distal to D15S122, two informative STS markers showed only a single maternal allele, suggesting that a recombination event occurred between D15S122 and GABRA5 on the der(15) or the maternal homologue. Methylation analysis also showed evidence for two extra copies of the maternal band using the *SNRPN *probe (not shown). Array CGH analysis showed evidence for tetrasomy of the region bordered by BP2-BP3 with no increased dosage telomeric to BP3 (Figure 2B) and a slight increase in dosage between BP1-2. By FISH, patient 00-16 carried two copies of a relatively small satellited idic (15) in each metaphase spread, each of which had two hybridization signals for the centromeric probe (Figure 2C). One of the normal chromosome 15s was also satellited. Both der(15) chromosomes hybridized with clones 437h9, 770c6 and 69i9 (Figures 2C–D), and no signals were detected with 204m06 (not shown). The presence of two der(15) chromosomes but only two extra copies as evidenced by array CGH and methylation analysis are consistent with a single extra copy of 15q11-q13 on each idic(15) chromosome, and suggest that the der(15) arose from an interchromosomal exchange involving BP2 and BP3.

### Patient 99.30

**Figure 3:**
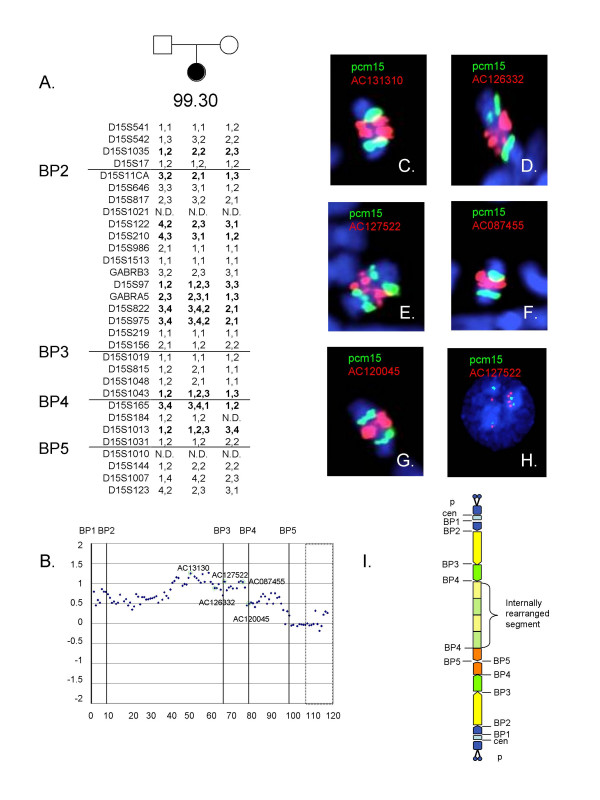
Molecular and cytogenetic data for patient 99.30. A. Genotyping with STS markers reveals evidence for an interchromosomal exchange leading to the formation of the der(15). Several STS in the proximal region between BP2-BP3 show only a single maternal allele, while distal to GABRB3 a biallelic maternal contribution is apparent. This region coincides with the area of increased dosage shown by array CGH (B). The array CGH data show tetrasomy of the region from BP1 that extends midway between BP2 and BP3. The distal end of this region through BP4 shows an increase in dosage to the hexasomy range. The interval between BP4 and BP5 is consistent with terasomy. The BP positions are indicated and the box indicates control probes. The positions of the clones used for FISH are highlighted. C-G. Metaphase FISH showing the der(15) chromosome indicated multiple signals for probes within the hexasomic region. Green signals are the centromeric probe pcm15. The BAC clones are indicated in red. H. Confirmation of hexasomy for clone AC127522 by interphase FISH with pcm15 (green) clone AC127522 (red). I. Schematic of the duplication chromosome for patient 99.30. The der(15) appears to have small satellites on each end. The der(15) extends to BP5 on each end but includes an two copies of an internally duplicated segment involving the distal portion of the BP2-BP3 interval and all of the BP3-BP4 interval indicated by the hatched yellow and green segments on the diagram. The position and orientation of the internally duplicated segment are not clearly defined but appear to be asymmetrically located within the der(15) based on the FISH data. In combination with two normal chromosome 15 homologs, this leads to hexasomy for the involved segments.

This subject was clinically diagnosed with an idic(15) chromosome described in the karyotype as 47,XX,+idic(15)(q13:q13).ish(CEP15++;SNRPN++). Examination of the genotypes for patient 99.30 revealed seven triallelic STS markers, five of these (D15S97, D15S822, D15S975, D15S1043, D15S1013) showing evidence for a maternal origin of the duplication chromosome. Proximal to D15S97, however, five informative loci (D15S542, D15S1035, D15S11, D15S122, D15S210) carried only a single maternal allele (Figure 3A). Methylation analysis showed an increase in dosage for the maternal band corresponding to two extra copies and consistent with the maternal imprinting of the der(15) (not shown). The array CGH results revealed evidence of a complex rearrangement with dosage consistent with tetrasomy from BP1 through clone AC012060, which spans nucleotides 23,923,383–24,087,529 on chromosome 15q12 between the ATP10A and GABRB3 genes. The log_2_T/R ratio for the overlapping distal clone, AC080121 (nt 23,936,750–24,091,741) increased to the hexasomy range and the increase in dosage extended to BP4. Between BP4 and BP5, dosage returned to the tetrasomic range (Figure 3B). FISH was performed on metaphase chromosomes for case 99.30 using probes that identify sequences along the proximal long arm (Figures 3C–H). On metaphase chromosomes, two clear paired signals were present for FISH proximal BP2-BP3 probes 770c6, 437h9, and no signal was present on the der(15) for probe 184n23 (not shown). Further analyses using probes within the area of increased dosage on the array (AC131310, AC128332, AC127522, and AC120046) recognized numerous signals on the der(15), consistent with the array CGH result (Figure 3C–G). Interphase FISH was performed using clone AC127522 in combination with pcm(15), revealing four signals on the der(15) for this probe in addition to the two normal homologues (Figure 3H). Thus, the patient has a partial hexasomy for the region of chromosome 15 that includes the GABA receptor cluster.

### Patient 03.46

**Figure 4:**
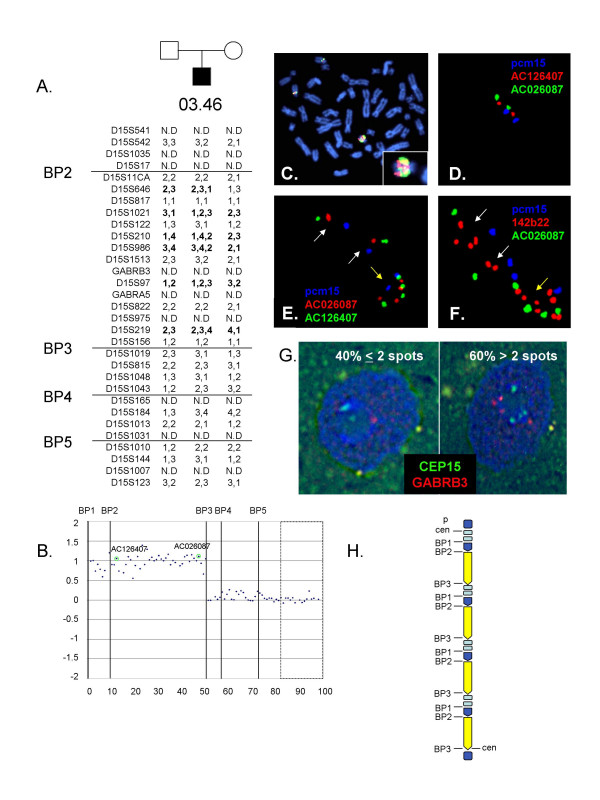
Molecular and cytogenetic data for patient 03-46. A. Genotyping for the family of patient 03.46 shows biallelic maternal contribution for alleles proximal to BP3. Informative loci beyond BP3 have only a single maternal allele (D15S1019, D15S1043, D15S184). B. Array CGH shows an overall dosage from BP1-BP3 in the tetrasomic range. The dotted box indicates control probes and positions of the fish clones are indicated. C. Metaphase FISH using probes 142b22 (red) and AC026087 (green) identify the two normal homologues as well as a large der(15) with large confluent signals for the 142b22 and AC026087 probes (inset). D and E. Interphase FISH with the centromeric probe, pcm15 (blue), AC126407 (red) and AC026087 (green) identifies two cell lines, one euploid cell line (D) and one that carries a der(15) chromosome (yellow arrow) in addition to the two normal homologues (white arrows). The der(15) is dicentric and includes four signals for each of the BAC clones. F. Hybridization of interphase nuclei for 03.46 with pcm15 (blue), 142b22 (red) and AC024087 identifies two normal homologues (white arrows), one of which shows an expansion of the NF1/IgH pseudogene region with four signals from the 142b22 probe. The der(15) shows two centromeric signals, and eight signals from the 142b22 probe and four signals from the AC026087 BAC clone. Ordering of the signals suggests a head to tail arrangement for the duplicated segments within the der(15). G. Tissue section from 03-46 (case SS-99-5552) frontal cortex analyses by FISH for CEP15 (green) and GABRB3 (red). Nuclei were counterstained with DAPI (blue). Mosaicism of der(15) was observed in brain nuclei, with 60% of nuclei showing >2 spots per nucleus for CEP 15 or GABRB3 and 40% of nuclei showing ≤2 spots per nucleus for CEP15 and GABRB3. Homologous pairing of 15q11-q13 alleles was observed around the nucleolus in most nuclei, making the counts of FISH signals range from 1–4 spots per nucleus for CEP15 and 1–6 spots per nucleus for GABRB3 out of a total of 149 nuclei counted.

The karyotype defined clinically for this subject was 47,XY,+idic(15)(q12;q12).ish(D1521+), with no annotation of mocaicism. Genotyping of the family of patient 03.46 revealed a biallelic maternal contribution for five STS markers proximal to between BP2 and BP3, with only single maternal alleles present beyond BP3 (Figure 4A). Methylation at SNRPN also was consistent with a maternal origin of the duplication chromosome (not shown). The log_2_T/R ratio on the array CGH revealed an overall tetrasomic dosage for the region and confirmed that the duplication extended to BP3 (Figure 4B), however metaphase FISH revealed mosaicism for a large dicentric chromosome that was present in 143 of 200 nuclei in PHA stimulated lymphocytes. Metaphase and interphase FISH studies of the duplication chromosome using probes pcm15, 142b22, AC126407, AC026087 revealed a complex dicentric chromosome that included four copies of probes AC126407, AC026087 and also had an expansion of the NF1/IgH pseudogene region detected by clone 142b22, which lies proximal to BP1 (Figures 4C–F). This expansion was present on one of the two normal homologues in both the euploid or aneuploidy cell lines. FISH on the parental cell lines identified the expansion on one of the maternal homologues (not shown). Interestingly, interphase FISH using the 142b22 probe in conjunction with centromeric probes and AC026087 demonstrated that the region proximal to BP1 was present within each of the duplicated segments, such that the der(15) appeared to include four head to tail copies of the NF1/IgH pseudogene region through BP3, flanked by two centromeres (Figure 4F). H. Schematic of the der(15) with light blue boxes indicating the NF1/IgH pseudogene region and duplicated segments indicated by yellow and blue arrows.

A fixed surgical sample of frontal cortex was available for this patient and was used for interphase FISH with probes CEP15 and GABRB3 (Figure 4G). These studies confirmed mosaicism in brain with 40.3% of cells showing less than or equal to two spots for the CEP15 and GABRB3 probes. Pairing of the signals was also examined. Pairing of the GABRB3 loci near the nucleolus was evident in both euploid and aneuploid neuronal nuclei, although some extra GABRB3 signals remained unpaired in der(15) containing nuclei.

### Family 99.10

**Figure 5:**
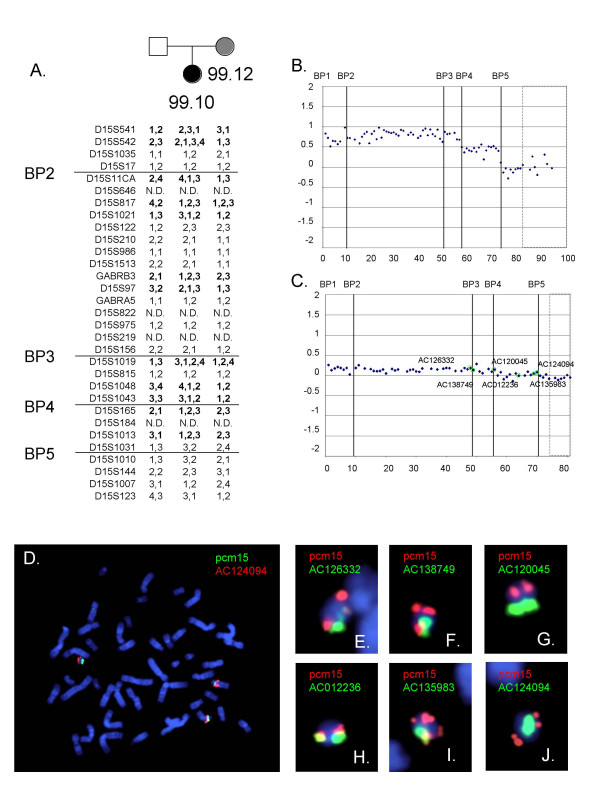
Molecular and cytogenetic analyses for 99.12, the mother of patient 99.10. A. Genotyping of the proband and parents identify a number of STS markers proximal to BP4 that are triallelic for the mother and tetra-allelic for the proband. The additional alleles suggest that the r(15) extends at least through BP4 and is present in the mother and proband. B. Log_2_T/R ratios for the array CGH for the proband demonstrate tetrasomy for the region between BP1-BP4 and trisomy for the region between BP4 and BP5. This is consistent with a der(15) that arose from a BP4:BP5 exchange. C. Array CGH using peripheral white blood cells from the mother indicates a slight increase in dosage for clones between BP1 and BP5 (spots 1–73). The mean log_2_T/R ratio was 0.108 (s.d. 0.060) compared with a mean ratio of 0.072 (s.d 0.065) (p = 0.037; two tailed unpaired t-test). For both arrays, the boxed area indicates control probes. The positions of the BAC clones used for FISH are indicated. D. Metaphase chromosomes hybridized with pcm15 (green) and BAC AC120045 (red) identifies two normal homologues and a small der(15) chromosome. E-J. Metaphase spreads hybridized with pcm15 (green) and additional BAC clones spanning the duplication regions. Most cells showed a single large signal for the pcm15 clone, suggesting the r(15) was monocentric.

Cytogenetic studies for proband 99.10 indicated that the patient had a complex karyotype with a dicentric der(15) chromosome and a 14:15 translocation [47,XX,der(14)t(14;15) (p11;p11)pat,+idic(15)(q12). ish der(14)(D15Z-,D15Z1+,D14Z1/D22Z1+),ish idic(15)(D15Z++,D15Z1++,SNRPN++)]. The der(14) chromosome was paternally derived and thought to be a normal variant. The clinically normal mother was found to be mosaic for a ring chromosome 15 [r(15)] which was present in approximately 50% of her peripheral white blood cells [maternal karyotype 47,XX,+r(15)(p11;q11.2) ish r(15)(D15Z1+,SNRPN+,AHT-)]. Examination of the genotypes for the family revealed evidence of a large ring chromosome in the maternal sample based on the presence of three alleles at D15S1021 and D15S1019, which lie between BP2-BP4. Additionally, the proband was found to be tetra-allelic for several STS markers, suggesting that she also carried the r(15) chromosome (Figure 5A), although it had not been seen in the clinical study. Array CGH was performed on samples from the patient 99.10 (Figure 5B) and her mother, 99.12 (Figure 5C). For the proband, dosage for the proximal long arm based on the Log_2_T/R ratios were typical for an idic(15) generated by a BP4:BP5 exchange[13], with no additional increase in dosage apparent despite the mosaic r(15). For the maternal sample, there was a subtle but significant increase in dosage for clones between BP1-BP5 (spots 1–73), with a mean log_2_T/R ratio 0.108 (s.d. 0.060) compared with a mean ratio of 0.072 (s.d. 0.065) for distal and control clones (p = 0.0365) (Figure 5C). FISH studies performed on the proband showed a large idic(15) arising from a BP4:BP5 exchange that was not mosaic. In addition, she carried a ring chromosome in approximately 10% of PHA stimulated lymphocytes (not shown). FISH using lymphoblasts and PHA stimulated peripheral leukocytes from the mother was performed using the centromeric probe in combination with BAC clones AC138749, AC127522, AC120045, AC012236, AC135983 and AC124094 and pcm15, each of which showed single signals on the r(15) confirming the der(15) carried the region of the proximal long arm through BP5.

## Conclusion

The cases described here indicate a wide range of complexity of duplication chromosomes derived from chromosome 15. In patient 00.16, the two der(15) were dicentric and each appeared to carry one extra copy of the region between BP2-BP3. This was further supported by the dosage measurements derived array CGH, which were consistent with overall tetrasomy of the duplicated region. However using array CGH without cytogenetic analyses would have missed the structural anomalies for the der(15)s. The molecular mechanisms that generated these idic(15) chromosomes are speculated to involve an interchromosomal exchange during meiosis, with a subsequent segregation error early in development, leading to two apparently identical idic(15) chromosomes.

Cases 99.30 and 03.46 have internal rearrangements within the der(15) that led to partial hexasomy for the involved segments. For 99.30, the increase in dosage for the region within the BP3-BP4 region was clearly detected by the array CGH studies and subsequently confirmed using FISH. Notably, at diagnosis, the complexity of the der(15) was not identified because the FISH probe used detected only the proximal BP2-BP3 region that was not included in the hexasomic segment. Phenotypically, this child performs on the lower end of the cognitive spectrum by comparison with other cases of idic(15) and is microcephalic (OFC < 2^nd ^centile for age), which is not typical of the syndrome. The der(15) carried by 03.46 is distinctive in that it appears to include 4 tandemly arranged segments, each of which includes the region proximal to BP1. While this region is typically included twice in the region between the centromere and BP1 in a typical idic(15) chromosome, it is unexpected that it would be included in the internally rearranged segments, indicating that the mechanisms that gave rise to this der(15) involved unique recombination events that lie outside the LCR. Pairing of the der(15) chromosomes in neurons has not previously been described. The proximity of the duplication chromosomes with the normal homologues is consistent with maintenance of perinucleolar organization of 15q11-13, despite the lack of the ribosomal RNA gene clusters in the der(15) chromosome. Homologous pairing of 15q11-13 is predicted to be important for the maintenance of biallelic expression of GABA_A _receptor genes (Hogart et al, 2007), but the effect of der(15) chromosomes on GABA_A _receptor gene expression is currently unknown. Previous expression studies in lymphocytes that demonstrated maternal expression of the *UBE3A *gene on idic(15) chromosomes[14].

For the family of 99.10, the large r(15) that was identified in the mother likely contributed to the subsequent abnormal crossover during oogenesis that led to the formation of the idic(15) in her daughter. The mother in this family is phenotypically normal, which likely reflects her low level mosaicism and may also represent a paternal origin of the r(15). Because grandparental samples were not available, it is not possible to determine the origin of the r(15). Array CGH performed with the prior knowledge of the r(15) was able to detect the presence of the r(15) however the low level dosage increase would not be expected to be detected clinically.

In two probands, 00.16 and 99.30, a shift in biallelic and monoallelic maternal contributions were observed, with change occurring in the BP2-BP3 interval in a region that has previously been identified as a recombination hotspot on chromosome 15q11-q12 in females. For 00.16, this likely represents an recombination event on the normal maternal homologue, although this cannot be definitively confirmed based on morphology of the der(15) chomosomes. For case 99.30, this shift between monoallelic and biallelic maternal contribution occurs in the region where the internal rearrangement of the der(15) is detected, hence increasing the likelihood a complex interchromosome and intrachromosomal exchange was involved in the generation of the the idic(15) chromosome. In summary, the cases described here indicate that not all der(15) chromosomes arise through nonhomologous allelic recombinations mediated by LCR present within the chromosome 15q11-q13 region.

## Methods

### Subjects and cell lines

The subjects were recruited to the study after clinical diagnosis of duplications of 15q11-q13 by karyotype combined with fluorescence in situ hybridization (FISH) analysis. Peripheral blood samples were collected from the patients and their parents following informed consent using protocols approved by the Institutional Review Board at the University of California, Los Angeles and the Alfred I. duPont Hospital for Children. Lymphoblast cell lines were established by transformation with Epstein-Barr virus using standard techniques. For one patient (03.46), a paraffin embedded surgical sample of frontal cortex was available. Phenotyping was performed in the child's home using the Mullen Scales of Early learning to examine cognition[15], and the autism diagnostic interview-revised [16] and autism diagnositic observation scale-generic[17] for the diagnosis of autism. Additional clinical data were obtained from physical examination and medical record review.

### Genotyping

To determine the parent of origin and mode of recombination, haplotypes were generated using 31 microsatellite markers amplified from genomic DNA purified from peripheral blood samples or transformed cell lines (Table 2). Primer sequences were obtained through the Genome Database (GDB) and the Marshfield Center for Medical Genetics. The 5' end of the forward primers was labeled with an infrared dye (LiCor, Lincoln NE). PCR was performed in a 10 μl reaction containing 25ng of DNA, 10× Ampli Taq Gold buffer (Applied Biosystems, Foster City, CA), Taq Extender (Applied Biosystems) dNTP's (1.25 mM each, Invitrogen. Carlsbad, CA), primers (0.2 μM each), 1–8 mM MgCl_2_, and 0.5 U Ampli Taq Gold (Applied Biosystems). Initial denaturation was for 10 minutes at 95°C, followed by 15 cycles of denaturation at 95°C for 30 seconds, annealing at 66°–51°C for 30 seconds, extension at 72°C for 30 seconds followed by 18 cycles of denaturation at 95°C for 30 seconds, annealing at 50°C for 30 seconds, extension at 72°C for 30 seconds, and a final extension at 72°C for 10 minutes. PCR products were visualized on a 6.5% denaturing polyacrylamide gel in the LiCor 4200 DNA analyzer. Genotypes were scored manually for each family.

**Table 2:** STS markers used for genotyping

Interval	STS Markers
BP1-BP2	D15S541, D15S542, D15S1035, D15S17
BP2-BP3	D15S11CA, D15S646, D15S817, D15S1021, D15S122, D15S210, D15S986, D15S1513, GABRB3, D15S97, GABRA5, D15S822, D15S975, D15S219, D15S156
BP3-BP4	D15S1019, D15S815, D15S1048, D15S1043
BP4-BP5	D15S165, D15S184, D15S1013, D15S1031
Distal to BP5	D15S144, D15S1007, D15S123

### Methylation Analysis

The imprinting status of the extra genomic material was ascertained using Southern blot analysis with the *SNRPN *probe[18]. Genomic DNA from blood was isolated using a Puregene DNA isolation Kit (Gentra Systems, Minneapolis, Minnesota) and resuspended in TE pH 8.0. DNA (10 μg) was digested with XbaI and NotI, a methylation sensitive restriction enzyme. Digestions were resolved on a 0.8% TAE gel and then transferred onto Hybond N+ nylon membranes (Amersham Pharmacia, Piscataway, NJ). Following transfer, DNA was UV crosslinked onto the membrane. Probes were labeled by random oligonucleotide priming using the Prime-It Random Labeling Kit (Stratagene, La Jolla, CA) and α-^32^P-dCTP (Perkin-Elmer. Waltham, MA) and hybridized onto the blots using PerfectHyb Plus Hybridization Buffer (Sigma Aldrich, St. Louis, MO). Hybridized blots were exposed onto phosphorimager screens (Amersham Pharmacia) overnight and images were scanned on a Storm phosphorimager (Amersham Pharmacia). Analysis was performed using the ImageQuant software package (Amersham Pharmacia). Presence of the maternal (4.2 kb) and paternal band (900 bp) was used to exclude PWS and AS. After measuring the signal intensities of both the maternal and paternal band, a maternal/paternal ratio was derived to determine dosage of the *SNRPN *region and the methylation status of the duplicated genomic region.

### Fluorescence in situ hybrization (FISH)

FISH analysis was performed as previously described[19]. Metaphase spreads or interphase nuclei were prepared from lymphoblastoid cell lines or phytohemaglutinin (PHA) stimulated cultures of peripheral leukocytes. DNA from BAC or cosmid clones (Figure 1) from chromosome 15 was nick translated and co-hybridized with a centromere probe for chromosome 15 (pcm15) generously provided by Dr. Mariano Rocchi (University of Bare, Italy). Signals on the normal homologues and the der(15) chromosomes gave were scored visually. Hybridization was detected by epifluorescence using a Leica DM RXA2 microscope using OpenLab 3.1.3 software (Improvision, Lexington, MA) or (patient 00-16) using a Leica DMX microscope equipped with a CCD camera and IPLab software (Scanalytics, Vienna, VA). A minimum of 20 metaphase spreads was examined for each probe. In mosaic samples, at least 100 nuclei were counted.

### Brain tissue FISH

Paraffin-embedded fixed frontal cortex from case 03.46 (ID: SS-99-5542) was sectioned at 5 μm onto glass slides. Slides were baked overnight at 56°C, then placed in four 5 minute washes with xylene, then two 5 minute washes with 100% ethanol, then 1 hour in 95°C in antigen retrieval solution (DAKO). Slides were then post-fixed in Histochoice for 90 minutes, then washed 5 minutes in 1× phosphate buffered saline (PBS). Slides were dehydrated in 50%, 70%, 90%, 100% ethanol (10 minutes each), then dried at 50°C. A probe mixture containing 1 μl each probe (CEP15 and GABRB3, Vysis, Inc.), 2 μl ddH_2_O, 7 μl LSI/WCP buffer (Vysis, Inc.) was warmed to 37°C, then added to the slide, coverslipped, and sealed with rubber cement. Probe and cells were simultaneously denatured at 85°C for 2 minutes on a slide cycler (Hybaid). Slides were incubated overnight at 37°C, the washed in 50% Formamide/50% 2× SSC thrice for 5 minutes, 0.5× SSC for 5 minutes, and 0.5× SSC/0.1% IGEPAL for 5 minutes, all at 46°C, pH 7.6. 250 μg/ml RNAse was added to the slides, coverslipped and incubated at 37°C for 30 minutes, then 5 minutes in 1× PBS and air dried. Slides were mounted with 5 μg/ml DAPI in Vectashield (Vector Laboratories), coverslipped and sealed with nail polish. Slides were analyzed on an Axioplan 2 fluorescence microscope (Carl Zeiss, Inc, NY) equipped with a Sensys CCD camera (Photometrics, Tucson, AZ), appropriate fluorescent filter sets, and automated xyz stage controls. The microscope and peripherals were controlled by a Macintosh running IPLab Spectrum (Scanalytics, Vienna, VA) software with Multiprobe, Zeissmover, and 3D extensions. Images were captured for blue, green, and red filters at one edge of the specimen, then repeated at 0.4 micron sections through the depth of the tissue. Each image stack was digitally deconvolved to remove out-of-focus light using HazeBuster software (Vaytek, Fairfield, IA). Following haze removal, image stacks for each fluorophore were merged and stacked to create a two-dimensional image representing all of fluorescence within the section.

### Array Comparative Genomic Hybridization (Array-CGH)

Array CGH was performed for each patient using custom BAC arrays as described [13]. The log_2_Test/Reference (log_2_T/R) ratios were calculated for each array probe and plotted linearly. Dosage was determined using the standard curve previously reported [13]. Positions of the array probes were based on the April 2003 genome assembly.

## List of Abbreviations

AS: Angelman syndrome

BAC: Bacterial Artifical Chromosome

BP: breakpoint

CGH: Comparative Genomic Hybridization

Der(15): derivative chromosome 15

FISH: Fluorescence in situ hybridization

GABA: gamma butyric acid

GDB: Genome Database

Idic(15): isodicentric(15)

Int dup(15): interstitial duplication (15)

LCR: Low copy repeats

log_2_T/R: log_2_Test/Reference

PBS: phosphate buffered saline

PWS: Prader Willi syndrome

r(15): ring chromosome 15

## Authors' contributions

The molecular and cytogenetic studies for the subjects were performed by NW (array-CGH, genotyping) and AP (metaphase and interphase FISH). Cell lines and tissue culture work including preparation of DNA and slide preparation were done by JD and BM. ND was the study coordinator involved in subject recruitment, consenting and sampling. KT performed the FISH analyses on the brain samples. CS, JLS and MS were responsible for oversight of the project, data interpretation, preparation of the manuscript and compliance with Institutional Review Board for human subjects protection. All authors contributed to manuscript preparation.
